# Semaglutide for weight loss: unanswered questions

**DOI:** 10.3389/fendo.2024.1382814

**Published:** 2024-06-05

**Authors:** Ploutarchos Tzoulis, Stephanie E. Baldeweg

**Affiliations:** ^1^ Department of Metabolism & Experimental Therapeutics, Division of Medicine, University College London, London, United Kingdom; ^2^ Division of Medicine, University College London, London, United Kingdom

**Keywords:** obesity, semaglutide, GLP 1 analog, overweight, anti-obesity medications

## Introduction

Obesity, a chronic disease associated with significant morbidity (1)and excess mortality (2), has become a global epidemic with a significant impact on healthcare systems and society in general (2). Lifestyle interventions, the cornerstone of treatment for obesity, have limited long-term efficacy, with maintenance of weight loss being the main challenge (3–5). The history of anti-obesity medications (AOMs) has been marked by withdrawal of more than 20 medications for safety reasons (6). Amongst the earliest AOMs developed, amphetamine derivatives were used widely since 1950s, but most, except for phentermine, have subsequently been withdrawn due to their cardiovascular risk and abuse potential (7). In recent years, the most prominent examples of AOMs failures have been sibutramine, rimonabant, and lorcaserin which were withdrawn secondary to adverse cardiovascular events, psychiatric safety, and increased occurrence of cancer, respectively (6, 8). Finally, the example of benfluorex, a medication used off-label for weight loss which, despite several case reports of cardiac valvulopathy, was withdrawn after significant delay (9), has highlighted the importance of pharmacovigilance. Until recently, AOMs available in Europe; namely, orlistat, combined naltrexone and bupropion, and liraglutide, a daily injectable glucagon-like peptide 1 (GLP-1) agonist (1); were underutilised in clinical practice (10, 11)since they produced a mean placebo-subtracted weight loss of around 5% (12), lower than the weight loss required to ameliorate most weight-related compications (12, 13). Semaglutide, a weekly injectable GLP-1 agonist widely used for the treatment of type 2 diabetes mellitus (DM), has been approved since 2021 by the US Food and Drug Administration (FDA) and the European Medicines Agency (EMA) for chronic weight management in patients with obesity or body mass index (BMI) above 27 kg/m^2and^at least one weight-related condition. Its weight-lowering effect is based on appetite reduction and satiety increase through activation of GLP-1 receptors in the hypothalamus and the hindbrain (14, 15). In comparison to other available AOMs, semaglutide shows a considerable enhancement in efficacy, being the first medication safely producing a greater than 10% average weight loss over that attributable to lifestyle interventions (12). A comprehensive programme of phase 3 randomised clinical trials (RCTs), the Semaglutide Treatment Effect in People with Obesity (STEP) trials, has evaluated the effect of semaglutide use for weight management in people with overweight or obesity, reporting a mean placebo-subtracted percentage weight loss of 12.5%, with more than half of participants achieving weight reduction of 15% or more from baseline (12). The unprecedented efficacy of semaglutide for weight loss has generated widespread publicity and led to global drug shortages. However, numerous research questions have arisen in relationship to its optimal use.

## What is the optimal duration of semaglutide therapy for weight loss?

The longest available efficacy data for semaglutide as an AOM are derived from the STEP 5 trial, showing a durable, sustained, substantial weight loss over a 2-year study duration with mean reduction in body weight of 15.2% in semaglutide group compared to 2.6% in placebo group (16). Both reduction in weight and improvement in cardiometabolic parameters, such as HbA1c, blood pressure and lipid profile, reached a plateau after 60 weeks and were sustained with its continued use for another year (16). Bearing in mind that obesity is a chronic, progressive, relapsing disease (17), the optimal duration of semaglutide therapy for weight management remains to be determined and may need to be tailored to individual characteristics. Future studies are required to investigate the durability of weight loss and metabolic benefits over different lengths of therapy and in different subgroups.

## Is weight regain after semaglutide discontinuation predictable and preventable?

In general, any successful strategy to tackle the obesity epidemic should start with the recognition that the modern obesogenic environment plays a key role, necessitating a societal approach (18). Implementation of public health policies is essential, for example lowering the content of trans fatty acids in food, promoting healthier diets and regular physical exercise in the school setting, and providing subsidies for healthy food choices combined with imposing taxation on sugar-sweetened beverages (18). Increasingly popular ‘‘cafeteria’’ or ‘‘junk food’’ diets, consisting of ultra-processed foods which contain high levels of refined sugar and saturated fat, lead to hyperphagia and obesity (19), highlighting the need for initiatives, such as user-friendly nutritional labelling of food products in order to empower people with the right information to make healthier choices. At a personal level and with respect to pharmacotherapy, significant weight gain after semaglutide discontinuation has been reported in two studies, STEP 4 (20) and STEP 1 trial extension (21). Individuals who discontinued semaglutide after 20 and 68 weeks regained over the next year off-pharmacotherapy 50% and 63% of the prior weight loss, respectively (20, 21). It is unclear whether this trajectory of weight regain continues in the subsequent years and to which extent, if any, a final net weight loss is achieved. More rapid and greater weight regain was observed in individuals who had achieved greater weight loss (21) and also when lifestyle intervention was withdrawn, such as in the STEP 1 trial extension (21), compared with the continuing provision of lifestyle intervention, such as in STEP 4 (20). These findings highlight the need for real-life withdrawal studies to identify the predictors of the rapidity and magnitude of weight gain and investigate the effect of continuing counselling and lifestyle modification on the trajectory of weight regain (21). Different strategies to prevent or, at least, ameliorate this phenomenon should be explored, including down titration of semaglutide dose, enrolling people in specific lifestyle programmes after drug discontinuation, and adopting specific criteria for another treatment course of semaglutide in case of rapid weight regain.

## Can we predict response to semaglutide?

All STEP trials have reported a marked variability in semaglutide response with 32–39.6% of people being ‘‘super responders’’, achieving weight loss in excess of 20%, and a subgroup of 10.2–16.7% of individuals being ‘‘non-responders’’, reducing body weight by less than 5% from baseline (16, 20, 22, 23).

Two predictors of lower weight loss with semaglutide have been identified; the coexistence of type 2 DM, with a mean body weight decrease of 14.9% in those without DM (22) versus 9.6% in those with DM (24), and the male gender, with an average 8–9.3% weight loss in males compared to 14–16.2% in females (25). The great heterogeneity in semaglutide-related weight loss highlights the need for studies examining the predictive role of demographic characteristics (gender, ethnic origin, age), metabolic parameters (baseline BMI, glycated haemoglobin, fasting glucose, markers of insulin resistance, lipid profile), eating behaviours (levels of hunger, satiety, episodes of hyperphagia, cravings) and genotype for weight response to semaglutide. Emerging evidence suggests that phenotype-guided, pathophysiology-based use of AOMs may enhance weight loss outcomes of pharmacotherapy, recommending GLP-1 agonists as the treatment of choice for people with ‘‘hungry gut’’ phenotype who exhibit abnormal satiety with reduced duration of fullness and rapid gastric emptying (26). Precision medicine initiatives, integrating data from genetics, epigenetics and metabolomics as well as encompassing demographic, environmental and psychological factors, are currently in progress in order to stratify obesity into different phenotypes with distinct underlying physiology and different risk for future complications (27, 28). Future studies will determine whether personalised decision-making about obesity pharmacotherapy, based on predictive models and novel algorithms, could optimise therapeutic benefit and minimise risks (27, 29). The advent of several novel AOMs, expected to be approved for clinical use in the near future, suggests a key role for prediction tools in day-to-day care to identify non-responders to semaglutide who would benefit more from alternative pharmacotherapies, such as dual GLP-1 and glucose-dependent insulinotropic polypeptide (GIP) agonist (30) or combination of GLP-1 agonist an amylin analogue (31) or triple GLP-1, GIP and glucagon receptors agonist (32), or endoscopic procedures or bariatric surgery (27).

## Which BMI cut-off should be used to consider semaglutide prescription for weight loss?

BMI, widely adopted as the marker used to define overweight and obesity, remains the main eligibility criterion for semaglutide administration. However, BMI is not an accurate measure of adipose tissue in the body and has two major limitations to diagnose obesity in an individual (33); firstly, it cannot distinguish fat from lean body mass which requires the use of dual-energy X-ray absorptiometry or bioelectric impedance; second, it cannot provide information about body fat distribution which would be feasible with use of anthropometric indices, such as waist circumference (17). Semaglutide administration for weight management has been approved for BMI greater than 30 kg/m^2^, regardless of comorbidities, or a BMI above 27 kg/m^2^ with at least one weight-related complication, producing similar percentage weight loss across different categories of baseline BMI (34). Taking into account the high prevalence of obesity, the high cost of semaglutide and its substantial budget impact, state and private health insurance providers will use varying BMI cut-offs as the criterion for reimbursement. For example, recent guidance in the United Kingdom recommends semaglutide as an option for weight management in people with BMI of at least 35 kg/m^2^, or alternatively a BMI of at least 30 kg/m^2^ with at least one weight-related complication. Therefore, cost-effectiveness analysis is essential in order to determine the optimal BMI range, across other criteria, which would ensure the most appropriate use of limited healthcare resources. Future studies need to evaluate whether predictive models, incorporating various parameters, should replace BMI as predictors of those at the greatest risk of complications who would benefit most from weight loss (27).

## What is the optimal lifestyle intervention as adjunct to semaglutide use?

In general, all people with obesity should receive individualised medical nutrition therapy and engage in regular physical activity (35). Any of multiple nutrition interventions can be considered with personalisation and long-term adherence being essential for sustained weight loss (35). With regards to the weight loss outcomes in patients treated with semaglutide as an adjunct to standard lifestyle intervention (16, 20, 22, 36) they were similar with those observed in STEP 3 trial in combination with intensive lifestyle modification (23), questioning the additional benefit of the inclusion of an initial 8-week meal-replacement phase or intensive behavioural therapy (23). Two trials, starting with an initial 12-week intensive lifestyle intervention followed by liraglutide (37) or tirzepatide, a dual GLP-1 and glucose-dependent insulinotropic polypeptide (GIP) agonist (38), produced additive weight loss which approached the combined results of each intervention on its own (37, 38),, suggesting that sequential use may be superior to concurrent use (38). The semaglutide-induced physiologically driven reduction in calorie intake puts the scope and intensity of lifestyle intervention into question (38), highlighting the need for prospective head-to-head studies comparing sequential with concurrent use of semaglutide across intensive caloric restriction diet. Finally, prospective studies need to compare the weight loss outcomes of semaglutide in combination with different diet regimens, varying on macronutrient composition and meal frequency, for example time-restricted eating.

## What is the role of semaglutide before and after bariatric surgery?

Weight regain following bariatric surgery is common (39), with numerous studies showing that liraglutide leads to significant weight reduction in these individuals (40–43). Due to their exclusion from the STEP programme, there is lack of high-quality evidence about semaglutide use in post-bariatric surgery populations. However, three retrospective real-world studies support the effectiveness and good safety profile of semaglutide for the treatment of weight recurrence after bariatric surgery, as evidenced by a mean 6-month body weight reduction of 9.8–10.3% (44–46). Ongoing prospective studies, such as the BARI-STEP trial, aim to determine the role of semaglutide in individuals who have experienced insufficient weight loss or excessive weight regain following bariatric surgery (47, 48). Another potential, albeit untested yet, therapeutic application of semaglutide is that of neoadjuvant pharmacotherapy prior to bariatric surgery. Prospective studies are warranted to determine whether semaglutide use in the preoperative setting could improve patient outcomes and reduce complications of bariatric operations, especially in individuals with very high BMI exceeding 50 kg/m^2^. Finally, future research efforts should also be directed towards the combination of semaglutide with, rapidly evolving, endoscopic bariatric therapies, such as intragastric balloons or endoscopic sleeve gastrectomy (48).

## What is the efficacy and safety of semaglutide for weight management in patients with active psychiatric disease?

Psychiatric conditions and obesity frequently occur together and have a bidirectional relationship (49), highlighting the need to generate specific data about semaglutide use in this context. Mental illness and obesity share not only pathogenic pathways involving the immune and endocrine system, but also coping behaviours and sociodemographic factors (50). Finally, it is worth mentioning that peptidergic systems critical to the regulation of energy homeostasis are also involved in neurocognition, as it applies to neuropeptide Y (NPY) and melanin-concentrating hormone (MCH) whose manipulation affects both appetite and diverse range of cognitive functions (51). Several psychotropic medications induce weight gain, contributing to high prevalence of obesity in people with psychiatric diseases (52). In individuals treated with antidepressants, semaglutide seems to retain its efficacy, as suggested by a *post hoc* exploratory analysis of STEP trials reporting a semaglutide-induced clinically meaningful weight loss, regardless of baseline antidepressant use (52). Antipsychotic-induced weight gain (AIWG), a common problem leading to chronic complications, may respond favourably to semaglutide, based on a small case series of patients with metformin-refractory AAWG (53). The lack of effective treatment options highlights the need for RCTs evaluating the effect of semaglutide on weight and metabolic parameters in patients with iatrogenic weight gain, such as olanzapine- and clozapine-treated patients with schizophrenia (54). Since STEP trials excluded patients who had major depressive disorder or bipolar disorder or schizophrenia within last two years prior to study enrollment (52), prospective studies are urgently needed to evaluate semaglutide use in patients with active mental health illness. Besides the need for efficacy outcomes in this population, the neuropsychiatric safety of semaglutide warrants evaluation in light of postmarketing reports about suicidal thoughts and behaviours in patients receiving semaglutide. Recent findings from a retrospective cohort study of patients receiving anti-obesity pharmacotherapy, reporting that semaglutide was associated with a significantly lower risk for suicidal ideation compared to alternative, non-GLP-1 agonists, AOMs (55), provide some reassurance. However, prospective studies are warranted to confirm the neuropsychiatric safety of semaglutide.

## What is the long-term safety profile of semaglutide?

Semaglutide-induced gastrointestinal events, albeit common, are transient, mild to moderate in severity, include gallbladder-related events, primarily cholelithiasis, and lead to drug discontinuation in 5.9–7.7% of patients (22, 23, 20, 34, 36, 56). In line with high-grade evidence supporting the absence of a link of GLP-1 agonists with pancreatitis, pancreatic cancer, medullary thyroid cancer, or other malignant neoplasms (57–59), STEP trials have reported extremely low rates for acute pancreatitis, no cases of pancreatic cancer, and rates of malignant neoplasms similar with those in placebo group (16, 20, 22, 23, 36). Also, a meta-analysis of all semaglutide studies has excluded an association of semaglutide with an increased risk of any types of cancer (60). Contrary to these reassuring safety data, a cohort study of people receiving pharmacotherapy for weight management indicated a significantly increased risk for pancreatitis, bowel obstruction and gastroparesis in those treated with GLP-1 agonists (61), including a 5-fold and 9-fold increased likelihood to develop pancreatitis and gastroparesis in semaglutide-treated patients, respectively (61). Additionally, postmarketing reports of ileus in patients on semaglutide have led the FDA to add a warning about gastrointestinal ileus on the semaglutide label, but a causal association has not been proven. In view of the very low background incidence and long latency period, clinical entities, such as ileus or pancreatic cancer, may go unnoticed in premarketing studies, necessitating post-marketing surveillance with various databases in order to draw definitive conclusions about a possible link with rare events (59). Finally, in line with any weight-lowering intervention (62, 63), semaglutide leads not only to reduction in fat mass, but also to reduction in total lean body mass and bone mineral density (22). These risks reinforce the importance of developing strategies to preserve muscle mass which, besides weight-bearing exercise and strength training, may entail novel pharmacological agents (63), namely bimagrumab, an activin antagonist (64), or myostatin inhibitors. Given the nature of obesity as a chronic, relapsing, progressive disease process, studies with long follow-up in combination with pharmacovigilance activities employing databases are warranted to ensure long-term safety of semaglutide and any other AOMs.

## Conclusive remarks

In conclusion, the popularity of semaglutide has soared due to a combination of great efficacy and good safety profile. However, much remains to be learnt about the optimal use of semaglutide, highlighting the need for prospective studies, both controlled and real-world, exploring key unresolved issues, including its duration of treatment, predictors of response, appropriate type of lifestyle intervention, long-term safety profile and its use in specific settings. Semaglutide may herald a new era in the management of obesity, introducing widespread use of pharmacotherapy to meet a large unmet clinical need. Answering these questions will determine the extent of semaglutide use as part of the rapidly expanding armamentarium and facilitate personalised treatment of obesity.

## Author contributions

PT: Conceptualization, Writing – original draft, Writing – review & editing. SB: Writing – original draft, Writing – review & editing.
